# Airborne PM_10_ Decreases Ku80 Expression and Ku70–Ku80 Heterodimer Levels of the Non-Homologous End Joining Repair Pathway in Lung Epithelial Cells

**DOI:** 10.3390/ijms26188936

**Published:** 2025-09-13

**Authors:** Ericka Marel Quezada-Maldonado, Javier Ivan Lozolla-Ortiz, Miguel Santibáñez-Andrade, Rocío Morales-Bárcenas, Claudia M. García-Cuellar, Yesennia Sánchez-Pérez

**Affiliations:** 1 Subdirección de Investigación Básica, Instituto Nacional de Cancerología, San Fernando No. 22, Tlalpan, Ciudad de México CP 14080, Mexico; marelquezada0612@gmail.com (E.M.Q.-M.); msantrade@ciencias.unam.mx (M.S.-A.); mobarobiol@yahoo.com.mx (R.M.-B.); 2 Departamento de Biología Celular, Centro de Investigación y de Estudios Avanzados del IPN (CINVESTAV), Av. IPN No. 2508, Col. San Pedro Zacatenco CP 07360, Mexico; lozollao13@gmail.com; 3 Dirección de Investigación, Instituto Nacional de Cancerología (INCan), San Fernando No. 22, Tlalpan, Ciudad de México CP 14080, Mexico

**Keywords:** particulate matter, DNA repair, DNA double-strand breaks, non-homologous end joining repair, Ku80 protein, Ku70–Ku80 heterodimer

## Abstract

The global population constantly breathes particulate matter with an aerodynamic diameter of ≤10 µm (PM_10_)—a human carcinogen linked to lung cancer. Previous studies have indicated that PM_10_ causes DNA damage, including double-strand breaks (DSBs). In particular, DSBs are primarily repaired by the non-homologous end joining (NHEJ) pathway, which is essential for maintaining genomic stability; however, the effects of PM_10_ exposure on this pathway are unknown. To address this, A549 lung epithelial cells were exposed to 10 µg/cm^2^ of PM_10_ for 6, 12, and 24 h. We determined that DSBs increased with prolonged exposure, and an increase in the frequency of micronuclei was found. Despite the accumulated DNA damage, no changes in the cell cycle were observed. Reductions in the levels of the Ku80 gene and protein, as well as the Ku70–Ku80 heterodimer—which is essential for initiating NHEJ-mediated repair—were observed. Levels of Artemis (which is responsible for processing DNA damage) remained stable, while levels of the XRCC4 gene and protein (responsible for completing repair) decreased. We conclude that PM_10_ disrupts two key proteins in the NHEJ pathway, impairing the capacity for DSB repair. This could promote the accumulation of DNA damage and induce genomic instability, contributing to the development of cancer.

## 1. Introduction

Approximately 91% of the world’s population is exposed to high levels of different harmful compounds due to air pollution. Therefore, air pollution has become a major public health concern. Particulate matter (PM) stands out as the most common pollutant with toxicological effects [1]. In 2013, the International Agency for Research on Cancer classified both air pollution and PM as human carcinogens [2,3]. Epidemiological and toxicological studies have reduced the knowledge gap regarding the association between long-term exposure to PM and the development of different types of cancer; mainly lung cancer. The incidence and mortality of lung cancer have significantly increased and, most importantly, research has recently focused on explaining the development of this disease in never-smokers [4,5,6,7].

PM is classified according to its aerodynamic diameter into PM_10_ (≤10 µm), PM_2.5_ (≤2.5 µm), and PM_0.1_ (≤0.1 µm), and comprises a complex mixture of compounds including polycyclic aromatic hydrocarbons (PAHs; e.g., benzo(a)pyrene (BaP), benzo(g)perylene, and naphthalene), transition metals (e.g., copper, nickel, vanadium and iron), and biological agents (pollen, virus, bacteria, and endotoxins) [8,9,10,11]. Approximately 50% of PM_10_ is composed of particles of smaller fractions [12,13,14]. PM enters the body via inhalation, inducing multiple effects throughout the respiratory tract until some compounds and the smallest particles are deposited in the alveoli, crossing the blood–alveolar barrier and causing systemic effects [15,16]. Although the association between chronic PM inhalation and lung cancer has already been defined, the continuous generation of toxicological evidence remains essential to understand the pathophysiology and evolution of cancers associated with exposure to this pollutant.

PM can induce DNA damage through multiple mechanisms associated with its components, with the formation of PAH–DNA adducts, single-strand breaks (SSBs), and double-strand breaks (DSBs) being the main genotoxic effects [17,18,19,20]. In addition to inducing DNA damage, PM_10_ deregulates genes and proteins involved in DNA repair mechanisms; specifically in the nucleotide excision mechanism that repairs DNA adducts [20,21,22]. However, knowledge about the effects of PM_10_ on DNA damage repair pathways responsible for DSBs is limited.

The non-homologous end joining (NHEJ) pathway repairs approximately 80% of all DSBs. It is considered the most efficient mechanism as it is activated rapidly after damage, does not require a sister chromatid, and acts during all stages of the cell cycle, predominantly during the G1 phase. Therefore, the NHEJ pathway prevents mutations by avoiding the transmission of altered DNA to the next generation of cells [23,24]. The NHEJ pathway is divided into three stages. The first stage involves the recognition of DSBs, which is mediated by the Ku70 and Ku80 proteins. These proteins form a heterodimer that interacts with double-stranded DNA ends and recruits the DNA-PKcs enzyme, which maintains the stability of DNA by inducing the phosphorylation of the Serine-139 residue of the histone H2AX, thus forming gamma H2AX (ƔH2AX)—a marker of DSBs. In the second stage, Artemis acts as an endonuclease or exonuclease. Finally, in the third stage, Pol mu or lambda synthesizes the missing nucleotides and XRCC4/ligase IV joins the ends of the DNA strands [24].

Dysfunction of the NHEJ pathway significantly contributes to the accumulation of unrepaired or poorly repaired DSBs, which increases the risk of malignant transformation and the development of diseases such as cancer [25]. Studies of the effects of PM_10_ on the NHEJ pathway are essential as PM_10_ increases the formation of micronuclei, suggesting the presence of genomic instability [22,26] which could be explained by defects in DNA repair, including those associated with the NHEJ pathway. The objective of this work was to evaluate the temporal effects of PM_10_ on DSB formation, as well as its effects on gene expression, protein levels, and protein complex formation in the NHEJ pathway components of human pulmonary epithelial A549 cells. We used doxorubicin as a positive control of DSB formation, considering its potential to activate the NHEJ pathway [27,28].

We determined that DSBs persist over time in cells exposed to PM_10_ and are accompanied by an increase in micronucleus formation, suggesting an increase in genomic instability. Despite DNA damage, no significant alterations in cell cycle distribution were observed. Regarding the molecular responses to damage, a decrease in both the gene expression and protein levels of Ku80, as well as a reduction in the binding capacity of the Ku70–Ku80 DSB recognition complex were detected in PM_10_-treated cells when compared to control cells. Although no changes in Artemis protein levels were found, XRCC4 gene and protein levels were reduced, which could compromise the efficiency of the NHEJ pathway.

## 2. Results

### 2.1. PM_10_ Induces DNA Damage Accumulation Through DSBs Without Causing Cell Cycle Arrest

**Figure 1:**
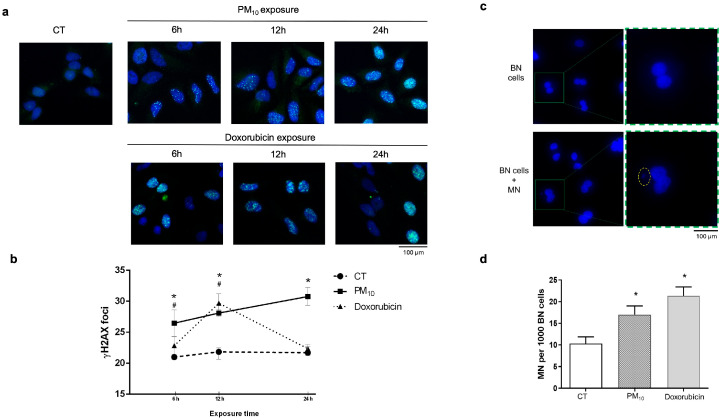
PM_10_ exposure induces double-strand break (DSB) accumulation and increase in micronuclei (MN) in A549 cells. DSBs were evaluated via immunofluorescence through the number of ƔH2AX (S139) foci in A549 cells exposed to 10 µg/cm^2^ of PM_10_ or 0.25 µg/mL of doxorubicin (positive control for DBS generation) for 6, 12, and 24 h. (**a**) A representative image panel of the combination of DAPI (core) and ƔH2AX (Alexa Fluor 488) foci at each exposure time and condition. (**b**) The number of ƔH2AX foci was counted from 100 individual cells and plotted on a line graph, showing the average number of ƔH2AX foci quantified per cell. Values represent the mean ratio plus error from three experiments. (*) indicates statistical differences of PM_10_ vs. control group; *p* < 0.05. (#) indicates statistical differences in doxorubicin versus control group; *p* < 0.05. (**c**) A representative panel of cells without MN and cells with MN. The square indicates a binucleate cell, which is magnified, the dotted oval identifies a micronucleus present in the binucleate cell. (**d**) Number of micronucleate (MN) cells after A549 lung epithelial cells were exposed to 10 µg/cm^2^ of PM_10_ or 0.25 µg/mL of doxorubicin for 24 h. The quantitative results are expressed as the proportion of MN cells, counting 300 events (cells) per condition. Values represent the mean ratio plus error from three experiments. (*) indicates statistical differences vs. control group; *p* < 0.05.

The formation of DSBs, as indicated by ƔH2AX foci, was analyzed after cells were exposed to PM_10_ or doxorubicin (Figure 1a). Compared to CT cells, there was a significant increase in the number of ƔH2AX foci in cells exposed to PM_10_ at 6 h (21.81 vs. 18.5; *p* < 0.05), 12 h (28.1 vs. 21.8; *p* < 0.05), and 24 h (30.77 vs. 21.7; *p* < 0.05), showing an increase with prolonged exposure. Doxorubicin-treated cells also showed an elevated number of ƔH2AX foci at 6 h (22.8 vs. 18.5; *p* < 0.05) and 12 h (30.36 vs. 21.8; *p* < 0.05) compared to CT cells. At 24 h, no differences were found in the number of ƔH2AX foci with respect to that in CT cells (Figure 1b).

The frequency of micronucleus (MN) formation in binucleated cells was measured at 24 h as an indicator of the persistence of DNA damage and genomic instability. PM_10_-treated cells showed a higher frequency of MN cells/BN cells when compared to CT cells (17.00/1000 vs. 10.33/1000 BN cells; *p* < 0.05). Doxorubicin-treated cells also showed a higher frequency of MN than CT cells (21.33/1000 vs. 10.33/1000 BN cells; *p* < 0.05) (Figure 1c,d).

### 2.2. PM_10_ Does Not Cause Cell Cycle Arrest Despite DNA Damage

**Figure 2:**
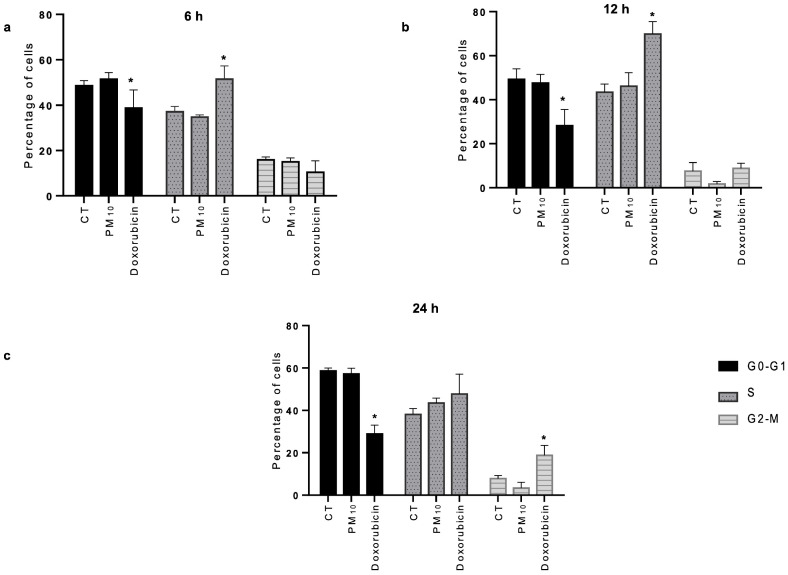
PM_10_ exposure does not cause alteration in the cell cycle in A549 cells. Cell cycle was evaluated via flow cytometry in A549 cells exposed to 10 µg/cm^2^ of PM_10_ or 0.25 µg/mL of doxorubicin for 6 (**a**), 12 (**b**), and 24 h (**c**). The percentage of cells in each cell cycle phase at each exposure time and condition are shown. Values represent results from three independent experiments, showing the mean ratio ± SD per treatment. (*) indicates statistical differences versus the control group; *p* < 0.05. In doxorubicin-exposed cells, the percentage of G1 phase cells significantly decreased compared to CT at 6 h (38.72% vs. 48.58%; *p* < 0.05), at 12 h (28.19% vs. 49.25%; *p* < 0.05), and at 24 h (28.86% vs. 58.63%; *p* < 0.05); the percentage of S phase cells significantly increased at 6 h (51.51% vs. 37.09%; *p* < 0.05) and at 12 h (69.80% vs. 43.41%; *p* < 0.05); and the percentage of G2/M phase cells was significantly increased at 24 h (18.76% vs. 7.86%; *p* < 0.05).

The cell cycle profile was analyzed and, despite the damage to double-stranded DNA, no significant changes were found in the cell cycle distribution in cells exposed to PM_10_ when compared to the CT cells at 6 h (Figure 2a), 12 h (Figure 2b), and 24 h (Figure 2c), with the G1 phase being predominant throughout the culture time. In the case of doxorubicin-exposed cells, the percentage of G1 phase cells significantly decreased compared to that in CT cells at 6 h (Figure 2a), 12 h (Figure 2b), and 24 h (Figure 2c), while the percentage of S phase cells significantly increased at 6 and 12 h as well as the percentage of G2/M phase cells at 24 h (Figure 2c).

### 2.3. PM_10_ Induces Changes in Gene Expression of Ku70 and XRCC4

**Figure 3:**
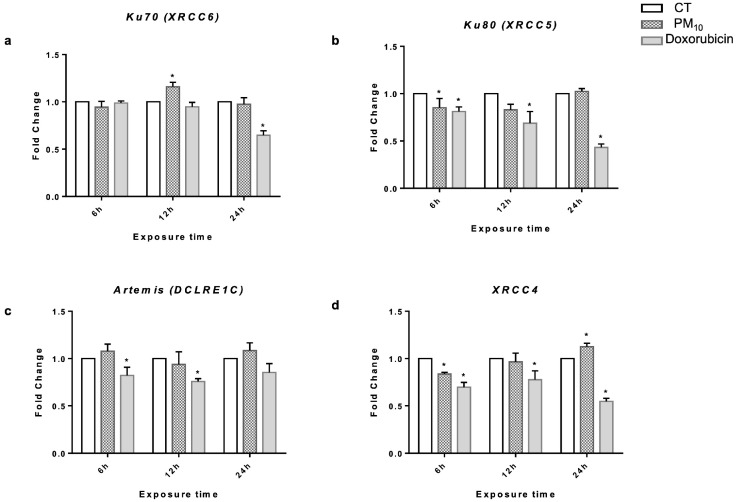
PM_10_ alters the expression levels of *Ku70*, *Ku80*, and *XRCC4* in A549 cells at different exposure times. The mRNA levels of (**a**) *Ku70* (XRCC6), (**b**) *Ku80* (XRCC5), (**c**) *Artemis* (DCLRE1C), and (**d**) *XRCC4* were evaluated via qRT-PCR in A549 lung epithelial cells exposed to 10 µg/cm^2^ of PM_10_ or 0.25 µg/mL of doxorubicin for 6, 12, and 24 h. The quantitative results were obtained using GAPDH as a housekeeping control for normalization. Relative quantification was performed, and the fold change (FC) was calculated using the 2^−ΔΔ Ct^ method. The values represent results from three independent experiments, showing the mean ± SD per treatment. (*) indicates statistical differences versus the control group; *p* < 0.05.

To determine whether cells respond to and repair DSBs after exposure to PM_10_, the expression levels of NHEJ genes were first measured. PM_10_-treated cells did not exhibit changes in *Ku70* (*XRCC6*) expression at 6 h when compared to the CT cells (Fold Change (FC) of 0.94 vs. 1.00). On the contrary, cells treated with PM_10_ for 12 h showed increased *Ku70* expression (FC of 1.16 vs. 1.00; *p* < 0.05) while, at 24 h, PM_10_ did not change the expression of *Ku70* (FC of 0.97 vs. 1.00). Doxorubicin-treated cells did not show changes in *Ku70* expression at either 6 h or 12 h (FC of 0.98, 0.94 vs. 1.00, respectively), while a decrease in its expression was observed in cells treated with doxorubicin at 24 h with respect to CT cells (FC 0.64 vs. 1.00; *p* < 0.05) (Figure 3a).

Regarding *Ku80* (*XRCC5*), PM_10_-treated cells showed a decrease in the expression of this gene when compared to CT cells at 6 h and 12 h (FC of 0.85, 0.80 vs. 1.00, respectively; *p* < 0.05), while no significant changes were observed at 24 h (FC 1.02 vs. 1.00). Doxorubicin-treated cells also showed decreased expression of *Ku80* at 6 h, 12 h, and 24 h (FC of 0.81, 0.68, and 0.43 vs. 1.00, respectively; *p* < 0.05) (Figure 3b).

No differences were observed in the expression of *Artemis* (*DCRE1C*) in PM_10_-treated cells at 6 h and 12 h with respect to CT (FC of 1.07, 0.93 vs. 1.00, respectively). On the other hand, cells treated with doxorubicin for 6 h and 12 h presented downregulation of Artemis (FC of 0.82, 0.75 vs. 1.00, respectively; *p* < 0.05). After 24 h of exposure, no statistical differences were found with respect to CT cells in PM_10_- or doxorubicin-treated cells (FC of 1.08, 0.85 vs. 1.00, respectively) (Figure 3c).

PM_10_-treated cells showed a significant decrease in the expression of *XRCC4* (*XRCC4*) compared to CT cells at 6 h (FC of 0.83 vs. 1.00; *p* < 0.05). After 12 h, treatment with PM_10_ did not change the expression of *XRCC4* (FC of 0.96 vs. 1.00). However, upregulation of *XRCC4* was observed in PM_10_-treated cells after 24 h (FC of 1.12 vs. 1.00; *p* < 0.05). Doxorubicin-treated cells showed decreased expression of this gene at 6 h, 12 h, and 24 h with respect to CT cells (FC of 0.69, 0.77, and 0.55 vs. 1.00, respectively; *p* < 0.05) (Figure 3d).

### 2.4. PM_10_ Decreases the Protein Levels of Ku80 and XRCC4

**Figure 4:**
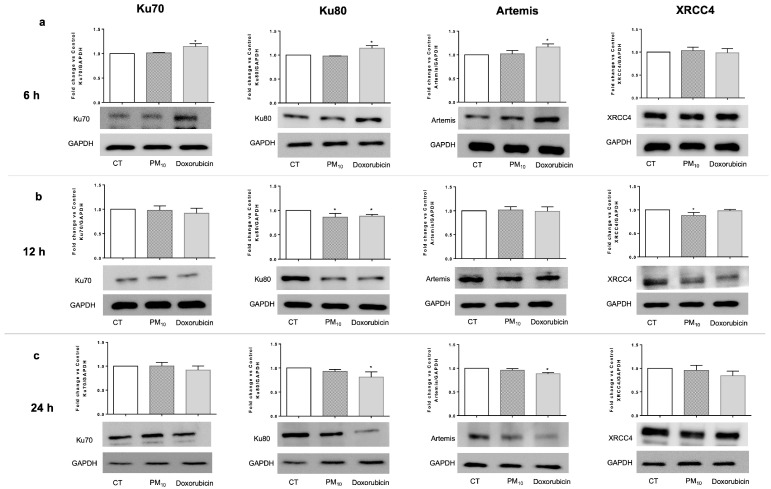
PM_10_ decreases protein levels of Ku80 and XRCC4, components of the NHEJ pathway, in A549 cells. The protein levels of Ku70, Ku80, Artemis, and XRCC4 were evaluated via Western blot (WB) in the total protein lysates of A549 lung epithelial cells exposed to 10 µg/cm^2^ of PM_10_ or 0.25 µg/mL of doxorubicin for (**a**) 6 h, (**b**) 12 h, and (**c**) 24 h. Representative images of WB (upper panels) and the densitometry analysis (lower panels) using GAPDH as a housekeeping control. The values represent results from three independent experiments, showing the mean ± SD per treatment. (*) indicates statistical differences versus the control group; *p* < 0.05.

Continuing to evaluate the NHEJ pathway, protein levels were measured after cells were exposed to PM_10_ or doxorubicin. Regarding the proteins that participate in the damage recognition stage, it was found that PM_10_-treated cells showed no alteration in Ku70 protein levels at 6 h, 12 h, or 24 h, whereas Doxorubicin-treated cells showed increased Ku70 protein levels at 6 h compared to CT cells (15%; *p* < 0.05). No differences were observed in this protein at 12 h or 24 h in cells exposed to doxorubicin. On the other hand, PM_10_-treated cells showed no modifications in Ku80 protein levels at 6 h but showed a decrease in this protein at 12 h when compared to CT cells (15%; *p* < 0.05). No difference was observed at 24 h. Doxorubicin-treated cells showed an increased Ku80 protein level compared to CT cells at 6 h (15%; *p* < 0.05), while it decreased at 12 h and 24 h (12% and 20%, respectively; *p* < 0.05) (Figure 4).

To analyze the damage processing stage, the level of Artemis was evaluated. It was found that PM_10_-treated cells did not exhibit any change in the level of this protein at either 6 h, 12 h, or 24 h with respect to CT cells. In contrast, doxorubicin-treated cells exhibited increased Artemis levels at 6 h (17%; *p* < 0.05) and a decreased level at 24 h (12%; *p* < 0.05), while no changes were observed at 12 h (Figure 4).

For evaluation of the final stage of DNA damage repair, XRCC4 was measured. No differences in protein levels were found in PM_10_-treated cells after 6 h and 24 h, while a significant decrease was determined at 12 h (12%; *p* < 0.05) when compared to CT cells. Doxorubicin-treated cells showed no differences in XRCC4 levels at 6 h, 12 h, or 24 h (Figure 4).

As decreased Ku80 and XRCC4 levels were detected at 12 h in PM_10_-treated cells, IF was performed to evaluate their cellular localization. It was found that cells treated with PM_10_ or doxorubicin exhibited no changes in sub-cellular localization for any of the analyzed proteins (Supplementary Figure S1). Furthermore, we confirmed that PM_10_ and doxorubicin treatments did not alter the Ku70 protein levels (Supplementary Figure S1a). In contrast, PM_10_ decreased Ku80 protein levels compared to CT cells (RFI of 0.83 vs. 1.00; *p* < 0.05); doxorubicin also decreased Ku80 protein levels at 12 h (RFI of 0.87 vs. 1.00; *p* < 0.05) (Supplementary Figure S1b). PM_10_ and doxorubicin-treated cells did not exhibit any change in Artemis protein levels at 12 h (Supplementary Figure S1c). Finally, PM_10_-treated cells showed decreased XRCC4 protein levels compared to CT (RFI of 0.81 vs. 1.00; *p* < 0.05), while no differences were observed in this protein after 12 h in doxorubicin-treated cells (Supplementary Figure S1d).

### 2.5. PM_10_ Decreases the Abundance of the DSB Recognition Complex

**Figure 5:**
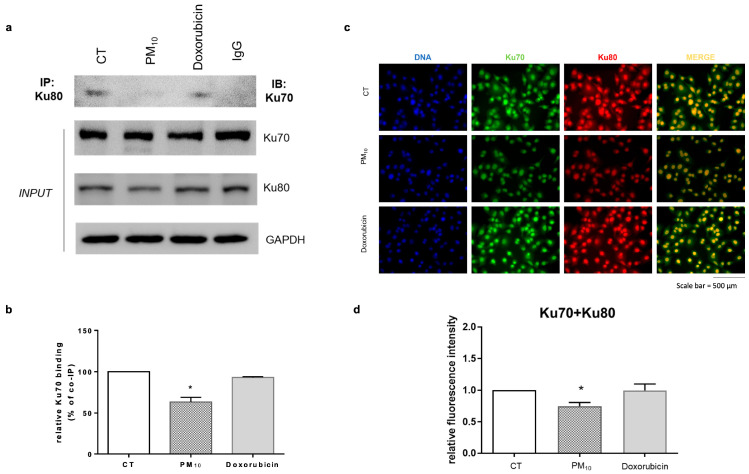
PM_10_ decreases the formation of the Ku70–Ku80 complex in A549 cells, likely preventing DSB repair. A549 lung epithelial cells were exposed to 10 µg/cm^2^ of PM_10_ or 0.25 µg/mL of doxorubicin for 12 h and (**a**) the formation of Ku70–Ku80 complex was analyzed via immunoprecipitation assay. A representative Western blot is shown (IP: immunoprecipitation, IB: immunoblot). (**b**) Percentage of binding of the Ku70–Ku80 complex, as calculated through densitometry analysis. (**c**) Representative images of co-localization between Ku70 and Ku80 detected via IF. (**d**) Densitometric analysis of IF. The values represent results from three independent experiments, showing the mean ± SD per treatment. (*) indicates statistical differences versus the control group; *p* < 0.05.

The recognition of DSB damage requires interactions between the proteins Ku70 and Ku80. Therefore, we evaluated the formation of the Ku70–Ku80 complex at 12 h after PM_10_ exposure via co-immunoprecipitation and fluorescence microscopy. PM_10_-treated cells showed a lower percentage of Ku70–Ku80 binding compared to CT cells (63% vs. 100%; *p* < 0.05) when measured via co-immunoprecipitation. In doxorubicin-treated cells, Ku70 co-immunoprecipitated with Ku80 at a similar rate as in CT cells (94% vs. 100%) (Figure 5a,b). In the same context, the co-localization of Ku70–Ku80 proteins analyzed via RFI demonstrated a decrease in the co-localization of these proteins after PM_10_ treatment compared to CT (0.74 vs. 1.00; *p* < 0.05). In doxorubicin-treated cells, there was no significant difference in Ku70–Ku80 interaction when compared to CT cells (0.99 vs. 1.00) (Figure 5c,d).

## 3. Discussion

DNA is constantly damaged by both endogenous processes and environmental agents. Among the forms of damage, DSBs are of particular concern due to their high capacity to induce genomic instability. To address these damages, cells rely on several repair mechanisms, with the non-homologous end joining (NHEJ) pathway being the most active and efficient throughout the cell cycle [29,30,31]. In this context, it is known that air pollution—and particularly particulate matter (PM_10_)—can cause DNA damage, including DSBs [19,32,33]. However, although there is evidence that certain components of PM_10_ affect DNA repair mechanisms [20,34], its specific impacts on the NHEJ pathway have not yet been fully characterized. Therefore, in this study, we set out to explore how PM_10_ affects key components of this repair pathway for the first time.

To address this, we used A549 lung epithelial cells—a widely validated pulmonary epithelial model in environmental toxicology [35,36,37]—and employed doxorubicin as a positive control for DSB induction and NHEJ pathway activation [38,39]. We observed a marked difference in damage dynamics: while cells treated with doxorubicin were able to reduce ƔH2AX foci at 24 h, cells exposed to PM_10_ maintained elevated levels of damage over time. This result suggests that PM_10_ compromises the cellular repair capacity. Different in vitro and in vivo studies have shown that cells can repair DNA damage induced by different agents within a few hours after treatment, as reflected by decreased levels of DNA damage markers such as ƔH2AX [40]. In this context, lymphoblasts (DT40 cells) exposed to hydrogen peroxide and lung fibroblasts (CCD-34Lu) exposed to radiation presented significant reductions in ƔH2AX foci at 24 h, indicating correct DNA repair [41,42].

In the same context, exposure to PM_10_ increased the frequency of micronuclei, which is consistent with previous studies reporting micronucleus formation following PM exposure [43,44]; as well as inducing DNA damage, which has previously been well documented through the comet assay [33,45,46], further supporting the genotoxic potential of these particles. The presence of these alterations in exposed cells suggests that the DNA damage caused by PM_10_ persists and is not efficiently repaired [47]. This phenomenon could be related to dysfunction in the NHEJ pathway, which is essential for the resolution of DSBs. Micronucleus formation is also a marker of genomic instability, reinforcing the hypothesis of deficient DNA repair in cells exposed to PM_10_. Although other DNA repair mechanisms—such as homologous recombination (HR)—could be involved in the resolution of DSBs, we consider their action insufficient in this context. Exposure to particulate matter simultaneously induces multiple types of genotoxic damage [17,48] which could saturate or interfere with repair pathways, thus preventing effective restoration of genomic integrity [49]. Additionally, we observed an increase in the frequency of micronuclei in cells treated with doxorubicin, even in the presence of reduced levels of γH2AX. This finding suggests that, in this case, micronuclei could arise from mechanisms alternative to direct DNA damage, such as centromere dysfunction or defects in mitotic spindle assembly [50,51,52].

Interestingly, no alterations or cell cycle arrest were observed in cells exposed to PM_10_ despite DNA damage, consistent with previous results [53]. This would indicate, on one hand, that DNA was damaged during all phases of the cell cycle; on the other hand, as the G1 phase predominated throughout the exposure time, the NHEJ pathway would be the main one for repairing DSBs [29,54]. An increase in the S phase followed by an increase in the G2/M phase was observed in doxorubicin-treated cells, as has been reported in different studies [55]. In general, cells with alterations in NHEJ repair genes or proteins tend to show a reduced repair capacity in all stages of the cell cycle [56,57].

To understand this phenomenon, we analyzed different components of NHEJ pathway. Several reports have indicated that increased levels of different NHEJ proteins can be detected as early as 4 h after the induction of DSBs [30]. In this study, we detected an increase in *Ku70* gene expression in cells exposed to PM_10_, but without a parallel increase in the associated protein. This could be due to dissociation between transcription and translation, or to post-translational mechanisms such as SIRT1 modulation, which is known to affect Ku70 levels and activity [58,59,60]; as such, it would be interesting to analyze SIRT–Ku70 complex formation during PM_10_ exposure. In parallel, both *Ku80* mRNA and protein decreased after exposure to PM_10_, thereby affecting the recognition of DNA damage.

Importantly, PM_10_ decreased Ku70–Ku80 heterodimer formation, which is necessary for the initiation of NHEJ pathway-mediated repair. This finding is crucial as this protein complex serves as a platform for the recruitment of other key players such as Artemis and XRCC4—the effector repair enzymes [61,62]. In cells exposed to PM_10_, we did not observe any changes in Artemis, but we observed reductions in the levels of the *XRCC4* gene and its protein, indicating that the pathway may be truncated from its early stages, impairing the recruitment of subsequent proteins. In contrast, in doxorubicin-treated cells, levels of Ku70, Ku80, and Artemis increased at 4 h of treatment, indicating DSB recognition [63] and correlating with reduced DNA damage, supporting the hypothesis that PM_10_ directly interferes with the NHEJ pathway.

Another interesting aspect is that our findings differ from previous studies evaluating the effects of individual PM_10_ components, such as PAHs [34,64]. These differences demonstrate that the use of PM_10_, a complex mixture of particles, is more representative of real-world environmental exposure. In this context, it is important to consider that the interactions between metals and organic compounds can generate synergistic or even antagonistic effects [11,65,66], resulting in different responses. In this context, several metals that are commonly present in PM_10_ are directly involved in alteration of the structures of repair proteins [67,68,69] or can modify them through mechanisms such as ubiquitination and proteasome degradation of proteins associated with oxidative stress [70,71,72]. Therefore, oxidant stress induced by PM_10_ [48,73,74] could be the principal mechanism inducing effects on the NHEJ pathway components. Furthermore, there is emerging evidence that some long non-coding RNAs (lncRNAs) and microRNAs regulate key components of the NHEJ pathway. LRIK and miR-145 interfere with NHEJ genes and heterodimer formation [75,76]. As PM_10_ has been shown to alter the expression of some non-coding RNAs [77,78], it is plausible to consider the possible participation of these molecules in NHEJ pathway deregulation.

It is important to consider that the PM_10_ analyzed in this study was collected exclusively in Mexico City—a city characterized by intense vehicular traffic and industrial activity. Previous studies have shown that urban PM_10_ contains higher concentrations of anthropogenic pollutants, such as PAHs and heavy metals, which are closely linked to genotoxic effects, when compared to elements present in rural areas that tend to induce less effects in DNA [45,79,80,81,82]. Therefore, future studies comparing the effects of PM10 from urban and rural environments on DNA repair mechanisms would be valuable for determining whether the cellular and molecular responses observed in this study are primarily driven by the specific toxic components present in urban air pollution. Such studies would also help to better assess broader health impacts in urban residents.

The disruption of the NHEJ pathway observed in this study could have significant implications for genomic stability and the malignant transformation potential of exposed cells. The persistence of unrepaired DNA damage is particularly concerning from a public health perspective, given that such damage can progressively accumulate in tissues subjected to chronic exposure to environmental pollutants, thereby increasing the risk of carcinogenesis [31,83]. Recent research has shown that prolonged exposure to PM_10_ can induce genetic mutations, a phenomenon that could be related to failures in DNA repair mechanisms [84]. In this context, future research should focus on elucidating the molecular mechanisms involved, including the roles of non-coding RNAs, oxidative stress, and other regulatory factors that could contribute to the alteration of genomic integrity maintenance systems under exposure to PM_10_. This study has some limitations that should be considered. First, the exclusive use of an in vitro model could limit the extrapolation of the results to more complex physiological conditions, where multiple cell types, immunological signals, and repair processes interact dynamically. Furthermore, exposure to PM_10_ was acute in this study, and it would be interesting to evaluate the effects of prolonged exposure, which is more representative of real-life pollution. Continuing to evaluate other DNA repair pathways could provide a more comprehensive understanding of the impacts of PM_10_ on these mechanisms, which would help better contextualize its carcinogenic effects.

## 4. Materials and Methods

### 4.1. PM_10_ Collection and Composition Characterization

PM_10_ was sampled in Mexico City during 2017 using nitrocellulose supports with 3.0 µm pore size (Sartorius, Goettingen, Germany) in a high-volume particle collector characterized by a constant flux of 1.13 m^3^/min (GMV model 1200 VFC HVPM10; Sierra Andersen, Atlanta, GA, USA). Collection was performed over a continuous 24 h period, three times per week. The collected PM_10_ was scraped from the nitrocellulose filters, and the samples were kept in endotoxin-free glass vials at 4 °C and protected from light [33,85]. The PM_10_ was characterized and a complete description of its elements, PAHs, and endotoxins has been provided in Santibáñez-Andrade et al., 2024 [11].

### 4.2. Cell Culture and PM_10_ Exposure

The lung epithelial cell line A549 was obtained from American Type Culture Collection (ATCC) and maintained in F12-K medium (Gibco 21127022, New York, NY, USA) supplemented with heat-inactivated fetal bovine serum (FBS) (10%) (Gibco, 16000044, Brooklyn, NY, USA) in a 5% CO_2_ atmosphere at 37 °C. The cells were seeded in culture plates and, 24 h later (70% confluence), were exposed to PM_10_ at a final concentration of 10 µg/cm^2^. The cells were resuspended and solubilized in F12-K culture medium supplemented with 10% FBS immediately before cell exposure. Importantly, this concentration has been reported as a sublethal concentration that does not affect cell viability [86,87], and is equivalent to five days of exposure in humans [79,88]. At the same time, cells treated only with F12-K medium (supplemented with 10% FBS) were maintained as control cells (CT), while those exposed to doxorubicin (Sigma-Aldrich, D1515, ST. Louis, MI, USA) at a concentration of 0.25 µg/mL were used as a positive control for DSB generation [38]. The effects induced by PM_10_ were evaluated after 6, 12, and 24 h of exposure.

### 4.3. Double-Strand Break Determination and Protein Localization via Immunodetection

A549 cells were grown on 8-well culture slides and exposed to PM_10_ (10 µg/cm^2^) and doxorubicin (0.25 µg/mL). The ƔH2AX foci (as an indicator of DSBs at 6, 12, and 24 h); the sub-cellular localization of the Ku70, Ku80, Artemis, and XRCC4 proteins; and Ku70–Ku80 protein complex formation (at 12 h) were analyzed via immunofluorescence. Following exposure, slides were washed with phosphate-buffered saline (PBS) and the cells were fixed with 4% paraformaldehyde at room temperature. To minimize autofluorescence, they were treated with fresh 0.1% sodium borohydride solution for 5 min. Subsequently, they were blocked with a solution containing 10% horse serum and 1% bovine serum albumin (BSA) in Tris buffer (TBS) for 1 h at room temperature, followed by washing with TBS. For protein detection, cells were incubated overnight at 4 °C with primary antibodies directed against ƔH2AX (Cell Signaling, 9718S; Danvers, MA, USA; 1:200), Ku70 (Cell Signaling, 4104; Danvers, MA, USA; 1:500), Ku80 (Invitrogen, MA5-1293; California, CA, USA; 1:500), Artemis (Invitrogen, PA5-102814; California, CA, USA; 1:500), and XRCC4 (Invitrogen, PA5-82264; California, CA, USA; 1:500) in 1% BSA-TBS. Cells were subsequently incubated with fluorochrome-conjugated secondary antibody (1:200) with anti-rabbit IgG (Jackson ImmunoResearch,115-544-144; West Grove, PA, USA) or anti-mouse IgG (Jackson ImmunoResearch, 115-585-146; West Grove, PA, USA) in 1% BSA-TBS for 45 min at room temperature in the dark. Coverslips were mounted on slides using DAPI to specifically stain nuclei. Immunofluorescence images were captured using an inverted microscope (Axio Observer Z1 DUO LSM 710 confocal system, Carl Zeiss; Oberkochen, Germany). The number of ƔH2AX foci (shown in green) were counted for 100 individual cells in each experimental condition, presented as the average number of foci. Relative fluorescence intensity (RFI) was obtained via densitometric analysis, using Image J 1.53 (Java 1.8.0 software, Bethesda, MD, USA) to compare the fluorescence intensity of the signal for sub-cellular localization of Ku70, Ku80, Artemis, and XRCC4, as well as co-localization of Ku70–Ku80 (observed in yellow) under experimental conditions (PM_10_ and doxorubicin) compared to their respective controls.

### 4.4. Micronucleus Assay

A549 cells were cultured in 8-well culture slides and exposed to PM_10_ (10 µg/cm^2^) or doxorubicin (0.25 µg/mL) for 24 h. Additionally, to prevent cytokinesis without interfering with karyokinesis, they were incubated with cytochalasin B (6 µg/mL; Sigma-Aldrich, C6762; St. Louis, MO, USA) for the same period. Subsequently, cells were fixed with a 3:1 ethanol and acetic acid solution and dehydrated through an increasing series of alcohols (70%, 90%, and 100%) for 2 min each. Slides were mounted using Pro-Long Gold Antifade with DAPI (Invitrogen, Carlsbad, CA, USA) for DNA counterstaining. Cytogenetic analysis was performed using an inverted microscope (Axio Observer Z1 DUO LSM 710, confocal system, Carl Zeiss, Oberkochen, Germany). A count of 1000 binucleated (BN) cells was performed for each experimental condition, and the proportion of cells with micronuclei (MN) was calculated.

### 4.5. Cell Cycle Analysis via Flow Cytometry

A549 cells were cultured in 6-well plates and exposed to PM_10_ (10 µg/cm^2^) or doxorubicin (0.25 µg/mL) for 6, 12, and 24 h. The medium was then removed, and the cells were washed with PBS. Mechanical dissociation with PBS containing 1 mM EDTA and each sample was performed. Then, the cells were fixed with 70% ethanol at −20 °C overnight. Samples were incubated with Trypsin 30 mg/mL at room temperature for 10 min. RNase A (0.1 mg/mL) and Trypsin Inhibitor (55 µg/mL) were added to the cells and incubated at room temperature for 10 min. Finally, cells were stained using 80 µL of propidium iodide (150 µg/mL), while protected from light. The cell cycle was analyzed via flow cytometry (BD FACS Canto II, San Jose, CA, USA), acquiring 20,000 events per condition. The analysis was performed using the ModFit LT software V. 3.3, BD, San Jose, CA, USA) 

### 4.6. RNA Analysis Expression via qRT-PCR

A549 cells were grown in 12-well plates and exposed to PM_10_ (10 µg/cm^2^) or doxorubicin (0.25 µg/mL) for 6, 12, and 24 h. The culture medium was then removed, and cells were washed with PBS. Total RNA was extracted using TRIzol reagent (Invitrogen, 15596026; Carlsbad, CA, USA), following the manufacturer’s instructions. RNA quantification was performed using an ND-1000 spectrophotometer (NanoDrop, Thermo Fisher Scientific; Wilmington, DE, USA), and only samples with an A260/280 absorbance ratio between 1.8 and 2.0 were used. cDNA synthesis was carried out using the High-Capacity cDNA Reverse Transcription Kit (Applied Biosystems, Thermo Fisher Scientific, 4368814; Foster City, CA, USA), in accordance with the manufacturer’s instructions. RT-qPCR was performed using the Real Q Plus 2x Master Mix Kit (Amplicon, A325406, Odense, Denmark), according to the manufacturer’s recommendations, using the StepOnePlus^TM^ Real-Time PCR System (Applied Biosystem, Thermo Fisher Scientific, 4376593, Foster City, CA, USA). Expression changes were analyzed using the 2^−ΔΔCt^ method, with GAPDH as an internal control. The oligos sequences for expression analysis were as follows: *XRCC6* (Ku70) (forward primer [Fwd] 5’-CCACAGGAAGAAGAGTTGGA-3’ and reverse primer [Rev] 5’- CTGCTCTGGAGTTGCCATGA-3’); *XRCC5* (Ku80) (Fwd 5’- CGACAGGTGTTTGCTGAGAA-3’ and Rev 5’ TCACATCCATGCTCACGATT-3’); DCRE1C (Artemis) (Fwd 5’-CCGCTTCGATAGGGAGAACC-3’ and Rev 5’ TCAAGCTGCACTCCAACCTT-3’); *XRCC4* (XRCC4) (Fwd 5’- GGCCTGATTCTTCACTACCTGA-3’ and Rev 5’- TAGCGGCTGCTGACTTGAAA-3’); *GAPDH* (GAPDH) (Fwd 5’-GCAAATTCCATGGCACCGTC-3’ and Rev 5’ AGCATCGCCCCACTTGATTT-3’).

### 4.7. Protein Level Determination via Western Blot

A549 cells were cultured in 6-well plates and exposed to PM_10_ (10 µg/cm^2^) or doxorubicin (0.25 µg/mL) for 6, 12, and 24 h. Protein levels were analyzed via Western blot (WB). Cells were washed with PBS and lysed using RIPA buffer (20 mM Tris-HCl pH 8; 150 mM NaCl; 1% NP-40), complemented with protease and phosphatase inhibitors (Thermo Fisher, 78441; California, CA, USA). Cell lysates were centrifuged at 12,000 rpm for 5 min at 4 °C, and supernatants were recovered for analysis. Protein concentrations were determined via bicinchoninic acid (BCA) assay using a bovine serum albumin standard curve (Thermo Fisher, 23209; California, CA, USA). A total of 20 µg of protein per sample was separated on 10% SDS-polyacrylamide gels at 150 V. Proteins were then transferred to membranes using a semi-dry transfer system (Trans-Blot Turbo, Bio-Rad, Hercules, CA, USA). For evaluation of Ku70, XRCC4, and GAPDH (control protein), 0.45 µm polyvinylidene difluoride (PVDF) membranes were used; while, for Ku80 and Artemis, 0.2 µm nitrocellulose membranes were used. Membranes were blocked with 5% albumin (BSA) in TBS-Tween 0.1% and agitated at room temperature for 1 h. Respective primary antibodies were incubated at concentrations of 1:1000 for anti-Ku70 (Cell Signaling, 4104; Danvers, MA, USA) or anti-XRCC4 (Invitrogen, PA5-82264; California, CA, USA); 1:500 for anti-Ku80 (Invitrogen, MA5-12933; California, CA, USA) or anti-Artemis (Invitrogen, PA5-102814; California, CA, USA); and 1:3000 for anti-GAPDH (Santa Cruz Biotechnology, 32233, California, CA, USA) overnight at 4 °C under constant agitation. Membranes were washed with TBS-Tween 0.1% and incubated with HRP-secondary anti-rabbit antibody (Amersham, NA934V; Buckinghamshire, UK, England) 1:3000 (Ku70, XRCC4, and Artemis) or HRP-secondary anti-mouse antibody (Amersham, NA931; Buckinghamshire, UK) 1:3000 (Ku80 and GAPDH) for 1 h. Finally, the membranes were incubated with chemiluminescent peroxidase substrate (Millipore, WBKLS0100; Burlington, MA, USA) and visualized using Chemidoc-It Imager UVP. To determine the changes induced by PM_10_ and doxorubicin, densitometric analysis was performed using the ImageJ software J 1.53 (Java 1.8.0 software, Bethesda, MD, USA).

### 4.8. Protein Complex Formation Analysis via Co-Immunoprecipitation Assay

A549 cells were plated in a 25 cm^2^ flask and exposed to PM_10_ (10 µg/cm^2^) or doxorubicin (0.25 µg/mL) for 12 h. Ku70–Ku80 protein complex interactions were evaluated via co-immunoprecipitation assay. After exposure, the cells were trypsinized and centrifuged at 1500 rpm for 5 min, and the obtained cell button was washed with PBS and centrifugated. The dynabeads Co-Immunoprecipitation kit (Invitrogen, Thermo Fisher Scientific, 14321D; Carlsbad, CA, USA) was used, according to the manufacturer’s specifications. The protein complex was obtained from 1 milligram of protein cell lysate immunoprecipitated with 1 milligram of magnetic beads using 10 µg of Ku80 antibody (Santa Cruz Biotecnology, California, CA, USA, 5280) or 5 µg of IgG antibody (IgG mouse, Santa Cruz Biotecnology, California, CA, USA, 2025). The Western blot for Ku70 (Cell Signaling, 4104; Danvers, MA, USA) was performed according to the steps described above. The percentage of binding of the Ku70–Ku80 complex was calculated through densitometric analysis using the ImageJ software J 1.53 (Java 1.8.0 software, Bethesda, MD, USA).

### 4.9. Statistical Analysis

The results are presented as the mean ± SD of at least three biological replicates (independent experiments). Statistical analyses were performed via one-way analysis of variance (Dunnett’s multiple comparisons post hoc test) using the GraphPad Prism Software, (version 6 for Windows, GraphPad Software, Boston, Massachusetts, USA). For MN cell frequency, a statistical analysis was performed using the χ2 test. A value of *p* ≤ 0.05 was considered statistically significant.

## 5. Conclusions

Exposure to 10 µg/cm^2^ of PM_10_ induced impairment of the NHEJ pathway in lung epithelial cells, as evidenced by downregulation of the Ku80 gene and protein, decreased Ku70–Ku80 complex formation, and reduced XRCC4 levels. Dysregulation of the NHEJ pathway—both at the damage recognition and end ligation stages—leads to the accumulation of DSBs and promotes micronucleus formation, both of which are characteristic markers of genomic instability. Impairment of the NHEJ pathway by PM_10_ may be an early event associated with carcinogenic processes.

## Data Availability

The original contributions presented in this study are included in the article/Supplementary Materials. Further inquiries can be directed to the corresponding authors.

## Supplementary Materials

The following supporting information can be downloaded at https://www.mdpi.com/article/10.3390/ijms26188936/s1.

## Abbreviations

PM	Particulate matter
NHEJ	Non-homologous end joining
DSBs	Double-strand breaks
IARC	International Agency for Research on Cancer
ƔH2AX	Phosphorylation at Ser-139 residue of the histone variant H2AX
