# Supplementary material for: Airborne PM10 Decreases Ku80 Expression and Ku70–Ku80 Heterodimer Levels of the Non-Homologous End Joining Repair Pathway in Lung Epithelial Cells
Source: Int J Mol Sci. 2025 Sep 13;26(18):8936. doi: 10.3390/ijms26188936 (PMC12469928; doi:10.3390/ijms26188936)
Supplement: Supplementary file 1 [file ijms-26-08936-s001.zip › ijms-3779127-supplementary.pdf]

Supplementary Materials:

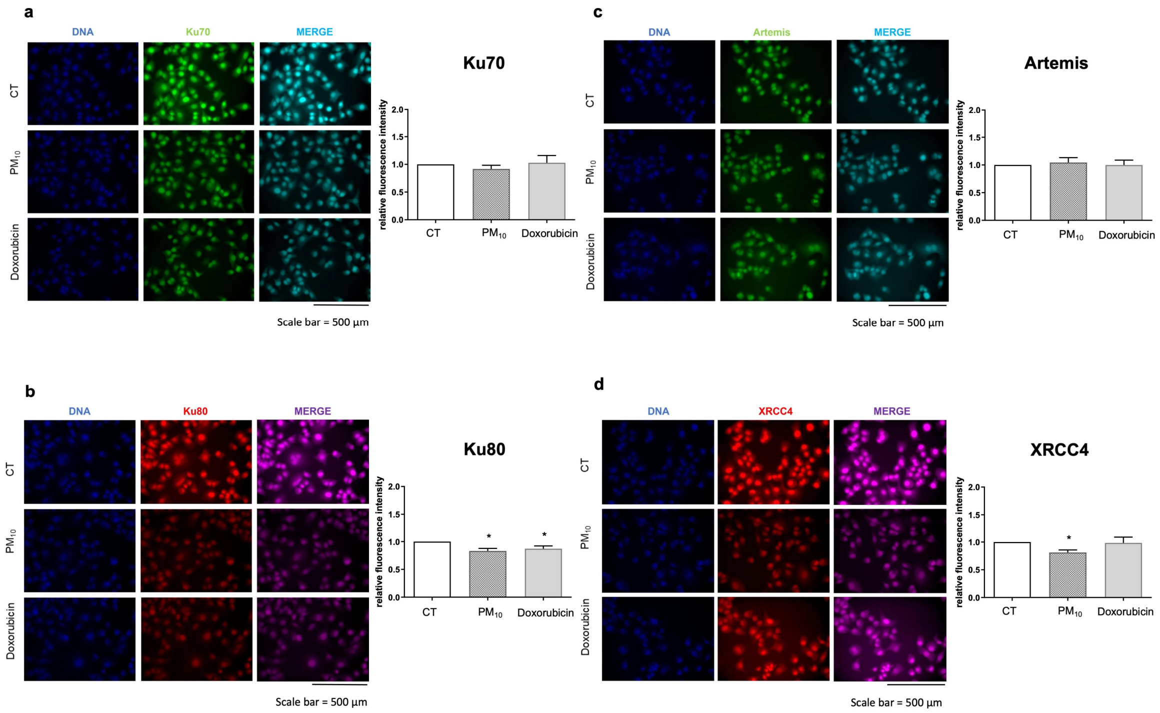

Supplementary Figure S1. *PM_{10} did not change the sub-cellular localization of the NHEJ pathway in A549 cells.* The protein localization and abundance of (a) Ku70, (b) Ku80, (c) Artemis, and (d) XRCC4 were evaluated by Immunofluorescence (IF) in A549 lung epithelial cells exposed to 10 µg/cm^2 of PM_{10} or 0.25 µg/ml of doxorubicin for 12 h. The image shows a representative panel of IF and the densitometry analysis. The values represent results from three independent experiments with the mean ± SD per treatment. (*) indicates statistical differences versus the control group; $p < 0.05$.
